# Two Strains of *Lactobacilli* Effectively Decrease the Colonization of VRE in a Mouse Model

**DOI:** 10.3389/fcimb.2019.00006

**Published:** 2019-01-30

**Authors:** Xianping Li, Liqiong Song, Siyi Zhu, Yuchun Xiao, Yuanming Huang, Yuting Hua, Qiongfang Chu, Zhihong Ren

**Affiliations:** State Key Laboratory for Infectious Disease Prevention and Control, Collaborative Innovation Center for Diagnosis and Treatment of Infectious Diseases, National Institute for Communicable Disease Control and Prevention - Chinese Center for Disease Control and Prevention, Beijing, China

**Keywords:** *Lactobacillus*, *Vancomycin-resistant Enterococcus*, fecal microbiota transplantation, 16S rRNA, RNA-seq

## Abstract

*Vancomycin-resistant Enterococcus* (VRE) infection is a serious challenge for clinical management and there is no effective treatment at present. Fecal microbiota transplantation (FMT) and probiotic intervention have been shown to be promising approaches for reducing the colonization of certain pathogenic bacteria in the gastrointestinal tract, however, no such studies have been done on VRE. In this study, we evaluated the effect of FMT and two *Lactobacillus* strains (Y74 and HT121) on the colonization of VRE in a VRE-infection mouse model. We found that both *Lactobacilli* strains reduced VRE colonization rapidly. Fecal microbiota and colon mRNA expression analyses further showed that mice in FMT and the two *Lactobacilli* treatment groups restored their intestinal microbiota diversity faster than those in the phosphate buffer saline (PBS) treated group. Administration of *Lactobacilli* restored Firmicutes more quickly to the normal level, compared to FMT or PBS treatment, but restored Bacteroides to their normal level less quickly than FMT did. Furthermore, these treatments also had an impact on the relative abundance of intestinal microbiota composition from phylum to species level. RNA-seq showed that FMT treatment induced the expression of more genes in the colon, compared to the *Lactobacilli* treatment. Defense-related genes such as defensin α, Apoa1, and RegIII were down-regulated in both FMT and the two *Lactobacilli* treatment groups. Taken together, our findings indicate that both FMT and *Lactobacilli* treatments were effective in decreasing the colonization of VRE in the gut.

## Introduction

*Vancomycin-resistant Enterococcus*(VRE), which was first reported in 1988, has become one of the most important nosocomial pathogens due to the administration of broad-spectrum antibiotics and immunosuppressive agents worldwide (Leclercq et al., 1988; Uttley et al., 1988). Most VRE infections are caused by *Enterococcus Faecium* (Paganelli et al., 2015). Antibiotic treatment causes destruction of the healthy gut microbiota and consequent loss of colonization resistance, which in turn promotes VRE growth and allows it to thrive and undergo expansion in the gastrointestinal (GI) tract. Although VRE intestinal colonization is asymptomatic, once traversing the intestinal epithelium barrier, it can lead to dangerous bloodstream infections in intensive care units (Caballero et al., 2017), endocarditis (Piszczek et al., 2015), and urinary tract infections (Mitchell et al., 2014; Piszczek et al., 2015). VRE is highly efficient in accumulation of antibiotic resistance genes and forms an important reservoir of resistance genes (Fremin and Bhatt, 2017). VRE-causing nosocomial infections are rising globally. Hospitalized patients infected with VRE increase the costs and number of days in hospital stays, and become a potential transmission source of VRE infection (Raven et al., 2017). Complete eradication of VRE from the gut is unlikely to ever be achieved. However, reducing VRE intestinal colonization can limit dissemination and systemic infection.

FMT is an effective and widely accepted therapy used in antibiotics-associated *Clostridium difficile* infections (Borody and Khoruts, 2011; Kelly, 2013; van Nood et al., 2013; Staley et al., 2017). Pamer et al. reported that both FMT (mainly obligate anaerobic bacteria) and the microbiota-containing *Barnesiella* can reduce VRE colonization of the GI tract in VRE-infected mice (Ubeda et al., 2013). FMT could eliminate both VRE and *Carbapenem-Resistant Klebsiella Pneumoniae* from the gut of the host (Caballero et al., 2015). However, application of FMT has potential challenges. For example, some donors may carry undetected pathogenic microorganisms, and some patients can't accept FMT psychologically. Therefore, alternative therapeutic strategies are urgently needed to prevent and control VRE intestinal colonization and dissemination. Probiotics are defined as “live microorganisms that, when administered in adequate amounts, confer health benefit to the host” (FAO, and WHO, 2001). Increasing evidence suggests that probiotics may be effective in improving the therapeutic effect in therapy of intestinal inflammation, cancer, obesity, and diabetes (Gao et al., 2015; Hudson et al., 2017; Routy et al., 2018; Zhao et al., 2018). *Lactobacillus* is a probiotic widely used in clinical setting. Compared to FMT, probiotics have clear constituents and controlled quality. It is reasonable to anticipate that some single strains of probiotics or consortium of probiotics would replace FMT in reducing VRE colonization. Indeed, probiotics have been proposed as an alternative strategy for decolonization treatment recently (Gao et al., 2015; Crouzet et al., 2018; Routy et al., 2018; Zhao et al., 2018). Toward this end, several clinical trials have evaluated the capacity of probiotics in eradicating VRE carriage; however, the results are inconsistent (Vidal et al., 2010; Szachta et al., 2011; Doron et al., 2015; Tytgat et al., 2016). This discrepancy may be due to differences in study protocols, in particular the different probiotics strains used. Therefore, we conducted this study to identify the ideal probiotic candidates that are effective in reducing pathogen colonization in VRE infection. Our results showed that the administration of two *Lactobacilli* strains could quickly decrease the number of VRE compared with PBS treatment with a VRE-lowering efficacy similar to FMT. We also analyzed different profiles of intestinal microbiota and colonic mRNA expression to determine the mechanisms involved.

## Materials and Methods

### Ethics Statement

This study was carried out in accordance with the recommendations of Animal management regulations (2017/03) and Measures for the Ethical Review of Biomedical Research Involving Humans (2016/12). The protocol was approved by the Ethics Review Committee of the National Institute for Communicable Disease Control and Prevention at the Chinese Center for Disease Control and Prevention.

### Screening and Cultivation of Probiotics Candidates

A total of 3 *Lactobacillus* strains isolated from feces of healthy breast-feeding infants and 17 *Lactobacillus* strains from feces of Tibet wild marmots without antibiotic pollution were included in this study. The *Lactobacilli* were isolated and screened using acid and bile salt tolerance assays. Briefly, one hundred microliter of feces diluted by serial 10-fold dilution were spread onto a Man-Rogosa-Sharpe(MRS) plate and then cultured in a carbon dioxide (CO_2_) incubator for 48 h at 37°C. *Lactobacilli* were identified via purification, 16S rRNA sequence and biochemical methods. Acid and bile salt tolerance assays were conducted by inoculating equal amounts of *Lactobacilli* into pH 2.0 MRS medium or regular MRS medium containing 0.3% bovine bile salt. After incubation in a CO_2_ incubator for 48 h at 37°C, the viable bacteria were counted. According to the results of salt and bile tolerance assays, we selected two candidate strains from infant and marmot fecal samples and named them Y74 and HT121, to further evaluate their efficacy in inhibiting VRE colonization in VRE-infected mice (Table S1). The two strains were washed with PBS and then re-suspended in sterile PBS at 1 × 10^9^ CFU/ml for inoculating mice.

### Mouse Model of VRE Infection

Female C57BL/6J wild-type mice (7-week old) were purchased from Beijing Vital River Laboratory Animal Technology Co., Ltd. All animal studies were performed in accordance with protocols approved by the Welfare & Ethical Inspection in Animal Experimentation Committee at the Chinese CDC. All mice were housed in a specific pathogen-free facility in the China CDC Animal Center. After 5 days of adaptation, all mice were randomly divided into 5 groups: control mice without any treatment, PBS-treated mice infected with VRE (PBS), FMT-treated mice infected with VRE (FMT), *Lactobacillus murinus* Y74-treated mice infected with VRE (Y74), and *Lactobacillus plantarum* HT121-treated mice infected with VRE (HT121). To infect animals, ampicillin (0.5 g/L) was added to drinking water. Seven days later, mice were intragastrically administered with 10^8^ CFU of the *Vancomycin-Resistant Enterococcus faecium* strain ATCC (700221) (a kind gift from Dr. Li Juan of Chinese CDC). One day after VRE infection, antibiotic treatment was stopped. Mice feces were collected daily for microbiota analysis using 16S rRNA high-throughput sequencing and VRE counts using culture on Enterococcosel agar plates supplemented with clindamycin (100 μg/ml), and vancomycin (40 μg/ml).

### FMT and Probiotics Treatments

Feces collected from 6 untreated healthy SPF C57BL/6J mice who shared one cage were homogenized in sterile PBS (2 fecal pellet/1 ml) and then cultured for 2 h under anaerobic conditions at 37°C. The culture suspension was filtered through 8-layer sterile gauze to remove sediment. One day after ampicillin treatment was stopped, 200 μl PBS alone, PBS containing fecal suspension, 5 × 10^8^CFU Y74,or 5 × 10^8^CFU HT121 was given to VRE-infected mice by oral gavage for 3 consecutive days (Ubeda et al., 2013).

### DNA Extraction, 16S rRNA Sequencing, and Data Analysis of Mice Intestinal Microbiota

Fresh feces were collected before mice were euthanized, frozen immediately, and stored at −70°C. DNA was extracted using the QIAamp DNA Stool Mini Kit according to manufacturer's instruction. DNA concentration was determined using the Qubit dsDNA HS assay kit or Qubit dsDNA BR assay kit in a Qubit Fluorometer or microplate reader. DNA integrity was measured with 1% agarose. The qualified samples were constructed into a database, sequenced, and analyzed by Beijing Genomics Institute Co., Ltd. The process is as follows: the V4 hypervariable region was amplified using modified universal bacterial primer pairs 515F (GTGCCAGCMGCCGCGGTAA) and 806R (GGACTACHVGGGTWTCTAAT) with Illumina adaptor overhang sequences. The V4 amplicon region was sequenced on the Illumina MiSeq PE250 platform and the raw data was pre-processed to acquire clean data. The consensus sequence was generated by FLASH (Fast Length Adjustment of Short reads, v1.2.11) (Magoc and Salzberg, 2011). The high quality paired-end reads were combined to tags based on overlaps, then tags were clustered into OTU (Operational Taxonomic Unit) by scripts of software USEARCH (v7.0.1090) with a 97% threshold (Edgar, 2013). Alpha diversity including the number of observed species, Chao1, and Shannon value are calculated by Mothur (v1.31.2) (Schloss et al., 2009). Histograms were generated using the software R (v3.1.1) at Phylum, Order, Class, Family, Genus, Species taxonomic level, respectively. LEfSe (http://huttenhower.sph.harvard.edu/galaxy) was used to determine significant differences between groups and threshold on the logarithmic LDA score for discriminative features 2.0. The original data can be downloaded from the NCBI SRA database (accession number: SRP 167776).

### Colonic RNA Extraction and RNA-seq Analysis

The colon was removed and stored in Trizol after mice were euthanized. Total RNA of colonic tissue was extracted using the Trizol agent according to the manufacturer's instruction, and total RNA concentration and integrity were determined using the Agilent 2100. The qualified samples were used to construct a library and sequenced on BGISEQ-500 by Beijing Genomics Institute Co., Ltd. The clean data was obtained from raw data by removing adapter-containing reads and more than 10% of N-containing reads and low-quality reads. The clean reads were aligned to the reference gene using Bowtie2 software and the reference genome using the HISAT software (Langmead et al., 2009; Kim et al., 2015). Gene expression quantification was determined by using the RSEM tool (Li and Dewey, 2011), and gene expressions were calculated using the FPKM method. For gene expression analysis, matched reads were calculated and normalized to FPKM using RESM software (Li and Dewey, 2011). Difference expressed genes (DEGs) were screened according to the default criteria with difference greater than or equal to 2-folds and deviation probability greater than or equal to 0.8. GO (Gene Ontology) has three ontologies, which describe the molecular functions, cellular components, and biological processes of genes, respectively. The analysis of GO was performed according to the Gene Ontology Database (http://www.geneontology.org/). The original data can be downloaded from the NCBI SRA database (accession number: SRP 167757).

### Quantitative Real-Time PCR

The genus of *Enterococcus* and *Lactobacillus* were validated by quantitative real-time PCR. DNA from the fecal samples was extracted using the QIAamp DNA Stool Mini Kit according to manufacturer's instruction. DNA samples were analyzed using quantitative PCR in an ABI 7500 Real-Time PCR System under the following conditions: initial denaturation at 94°C for 30 S, followed by 40 cycles of 95°C for 10 s, 56°C for 30 s. For genus *Lactobacillus*, the annealing temperature was 65°C. The number of the copies of the genera *Enterococcus* and *Lactobacillus* were calculated according to their standard curves. The primers and probes of the genera *Enterococcus* and *Lactobacillus* are listed in Table 1 (He and Jiang, 2005; Costa et al., 2014).

**Table 1:** Sequences of real-time PCR primers and probes.

**Genes name**	**Sequence**
*Enterococcus* genus	Forward 5′-AGAAATTCCAAACGAACTTG-3′
	Reverse 5′-CAGTGCTCTACCTCCATCATT-3′
	Probe 6-FAM-TGGTTCTCTCCGAAATAGCTTTAGGGCTA-BHQ1
*Lactobacillus* genus	Forward 5′-GTGGTGCGGTCGATATTTTAGTT-3′
	Reverse 5′-TCAGCCGCGCTTGTAACC-3′
	Probe 6-FAM-TTGACTCGGTGGCGGCCTTAGTGCCA-BHQ1
β actin	Forward 5′-GGCTGTATTCCCCTCCATCG-3′
	Reverse 5′-CCAGTTGGTAACAATGCCATGT-3′
Apoal	Forward 5′-GGCACGTATGGCAGCAAGAT-3′
	Reverse 5′-CCAAGGAGGAGGATTCAAACTG-3′
Defa2	Forward 5′-GACCTGCTCAGGACGACTT-3′
	Reverse 5′-CCTCAGAGCTGATGGTTGTCA-3′
Defa3	Forward 5′-TCGCTGAACATGGAGACCAC-3′
	Reverse 5′-CGAGGTAGTCATCAGGCACC-3′
Defa21	Forward 5′-CTCCTCTCTGCCCTCATCCT-3′
	Reverse 5′-GGCCTCCAAAGGAGACAGAC-3′

Some DEGs were validated by quantitative real-time PCR. Total RNA of colonic tissue was extracted using the Trizol and reverse-transcripted to generate cDNA by reverse transcriptase (Promega), according to the manufacturer's instructions. cDNA was analyzed using quantitative PCR in an ABI 7500 Real-Time PCR System under the following conditions: 95°C for 30s, then 60°C for 15s. 95°C for 15 s, followed by 40 cycles of 95°C for 10 s and 60°C for 32 s. A dissolution curve was run under the conditions: 95°C for 15s, 60°C for 60s. The folds in the DEGs were calculated using the comparative 2^−ΔΔ*Ct*^ method.

### Statistical Analysis

Results are presented as means ± SD. Statistical analysis was performed using one-way ANOVA with Tukey test (VRE counting in feces of VRE-infected mice, OTU numbers, Chao1, and Shannon index of mice fecal microbiota, mRNA expression of Differentially Expressed Genes in colonic tissue, the relative abundance of fecal microbiota at the level of the family, and genus) or Kruskal-Wallis test (for some skew distributional data of the relative abundance of fecal microbiota at the level of the family, and genus). Differences at *P* < 0.05 were considered significant.

## Results

### Treatments With Two *Lactobacilli* Strains and FMT All Reduced the Number of VRE in the Feces of VRE-infected Mice

**Figure 1:**
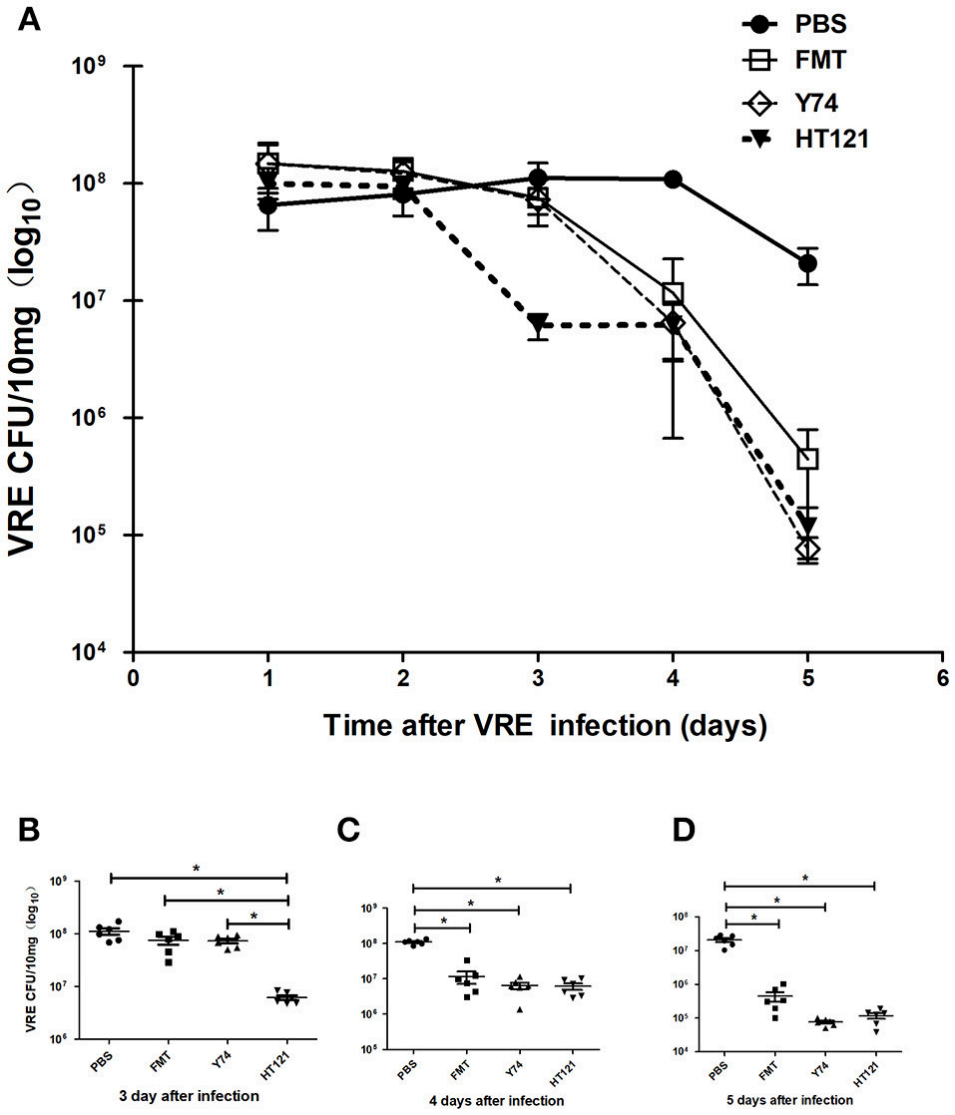
Treatments of *Lactobacillus murinus* Y74, *Lactobacillus plantarum* HT121 and FMT all decreased the colonization of VRE in VRE-infected mice. All mice were orally infected with 10^8^ CFU VRE and four different treatments were administrated 2 days after VRE infection. PBS, FMT, Y74, and HT121 represented the different VER-infected mice being treated with PBS, Fecal microbiota transplantation, *Lactobacillus murinus* Y74, and *Lactobacillus plantarum* HT121, respectively. **(A)** The kinetic counts of VRE in the feces of VRE-infected mice treated with PBS, FMT, Y74, or HT121, respectively, during the 5-day period of infection. **(B)** The colonization of VRE in different groups treated with PBS, FMT, Y74, or HT121 respectively on day 3 after infection. **(C)** The colonization of VRE in different groups treated with PBS, FMT, Y74, or HT121 respectively on day 4 after infection and **(D)** The colonization of VRE in different groups treated with PBS, FMT, Y74, or HT121 respectively on day 5 after infection. Data are means ± SD, *n* = 6 mice. *P < 0.05.

VRE-infected mice were treated with *Lactobacillus* Y74 or HT121, or FMT two days after VRE infection. After 1 day of treatment, the number of VRE in the feces of HT121-treated group was significantly lower than those in the other three groups (Figures 1A,B). After 2 days of treatment, the number of VRE in the feces of Y74, HT121 and FMT-treated groups were all significantly lower than that in the PBS-treated VRE group, while no significant difference for this effect was found among the three treatment groups (Figures 1A,C). Similar results were observed at day 3 post-treatment (Figures 1A,D).

### FMT, Y74, or HT121 Treatment Partly Restores the Diversity of Intestinal Microbiota in VRE-infected Mice

**Figure 2:**
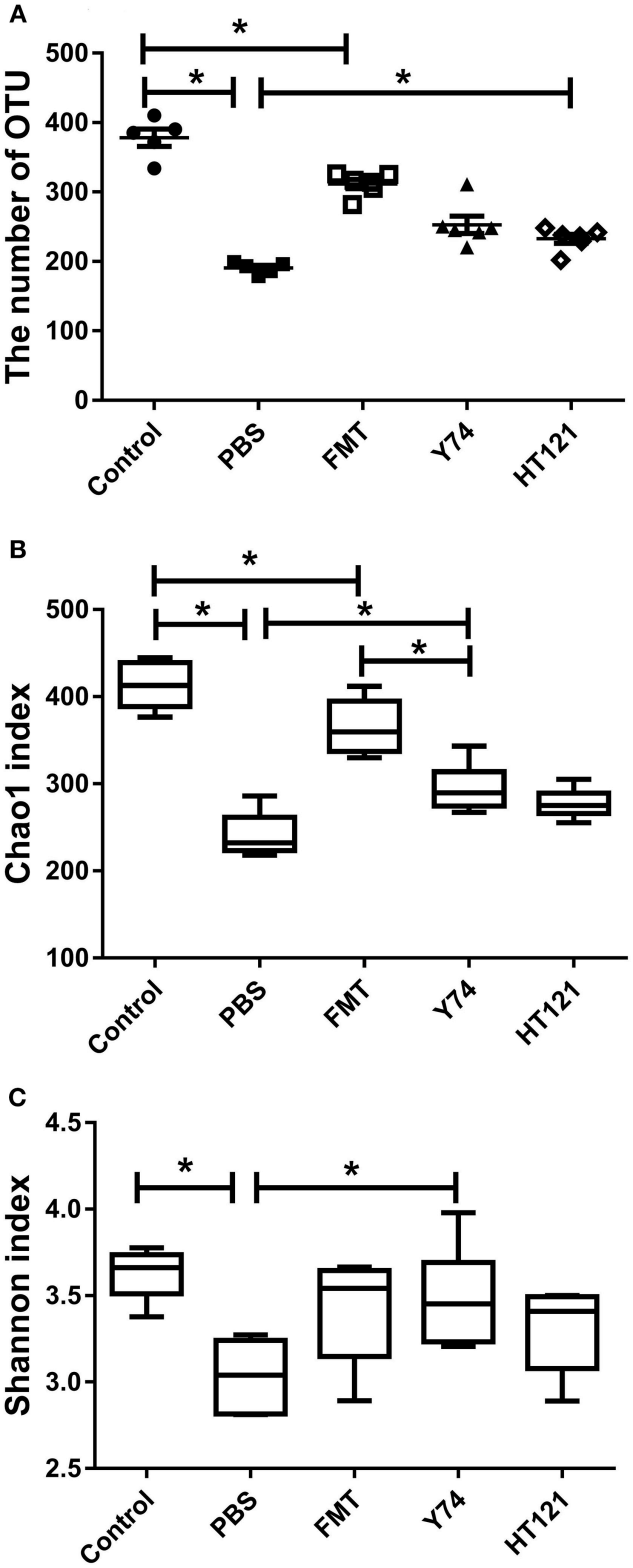
OTU numbers, Chao1, and Shannon index of mice fecal microbiota after 5 days after infection. **(A–C)** represent the OTU number, the Chao1 index, and the Shannon index, respectively. Control, PBS, FMT, Y74, and HT121 represent blank control group (uninfected group without any treatment), PBS-treated VRE group, FMT-treated VRE group, Y74-treated VRE group, and HT121-treated VRE group, respectively. *P < 0.05.

To explore possible mechanisms of the VRE-reducing effect induced by different interventions, we compared the differences in relative abundance of intestinal microflora composition among mice treated with Y74, HT121, or FMT for 3 days. We performed sequencing dilutions to ensure the depth of sequencing covered all species of the fecal samples (data not shown) and used the number of OTUs and the Chao1 index to measure the richness of intestinal microbiota. The OTU number and Chao1 index of all infected mice were significantly smaller compared to those in the uninfected mice (Figures 2A,B). All three treatments (FMT, Y74, HT121,) reduced the number of OTUs and the Chao1 index to various degrees. FMT treatment was more effective to improve the richness of intestinal microbiota, compared with Y74 or HT121 treatment (Figures 2A,B). We also used the Shannon index to measure the diversity of intestinal microbiota. A higher Shannon index indicates more abundant diversity of the species in a sample. Our data showed that the Shannon index of PBS group is significantly lower than that of control and Y74 groups, suggesting that VRE infection reduced the diversity of the microbiota and conversely, *Lactobacillus* and Y74 treatment partly restored the microbiota (Figure 2C).

### FMT, *Lactobacillus* Y74, and *Lactobacillus* HT121 Treatment Affects the Fecal Microbiota Composition at the Level of the Phylum and Class

**Figure 3:**
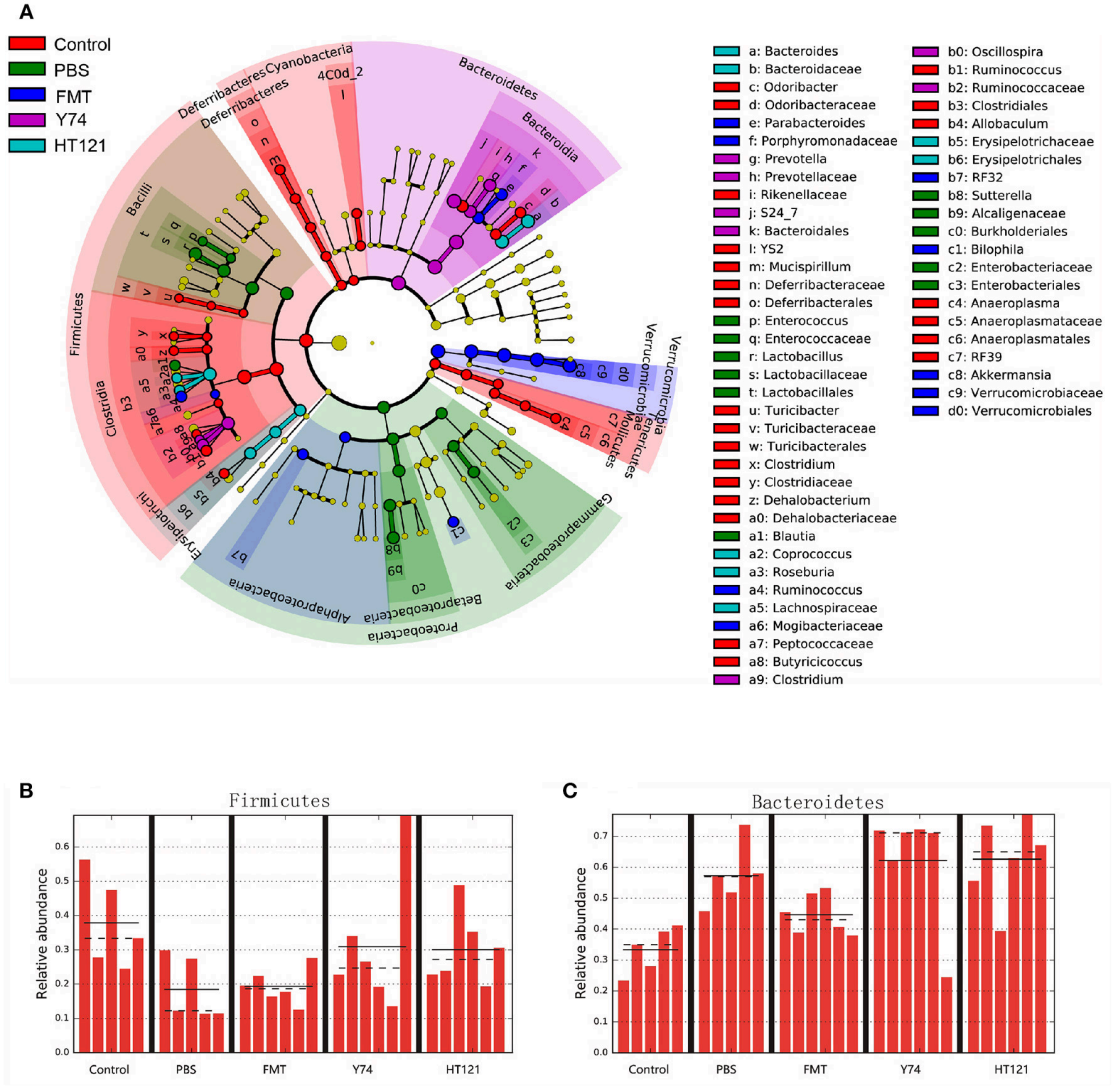
Lefse identified the difference taxa among five groups. Cladogram **(A)** and the relative abundance of Phylum of Firmicutes **(B)** and Bacteroides **(C)** from the Lefse analysis. Control, PBS, FMT, Y74, and HT121 represented control group, PBS group, FMT group, Y74 group and HT121 group, respectively.

16S rRNA sequencing analysis showed changes in the relative abundance of the fecal microbiota at all taxonomical levels from phylum to species after 3 days of intervention with PBS, FMT, Y74, or HT121. As shown in Figure S1 and Figure 3A, at the level of phylum, the main differences in relative abundance among the groups were found in Firmicutes, Bacteroidetes, Proteobacteria, and Verrucomicrobia. The relative abundance of Firmicutes in PBS-treated VRE group and FMT group were significantly lower than that in the uninfected group (*P* < 0.05), while Y74 and HT121 groups had similar levels of Firmicutes compared with the uninfected group (Figure 3B). The relative abundance of Bacteroidetes in PBS, Y74, and HT121 groups were significantly higher than that in the uninfected group (*P* < 0.05). Only FMT treatment decreased Bacteroidetes to a level close to that seen in the uninfected group (Figure 3C). The relative abundance of Proteobacteria accounted for a small proportion of the variation. Antibiotic pretreatment and VRE infection resulted in higher levels of Proteobacteria in all infected mice, and HT121 treatment restored Proteobacteria to levels similar to those in the uninfected mice, but Y74 and FMT had no such effect (Figure S2A). Antibiotic pretreatment and VRE infection induced higher levels of the families Enterobacteriaceae and Sutteralla, which were restored by FMT, Y74, or HT121 treatment to normal levels (Figures S2C,D). Both Y74 and HT121 treatments significantly decreased the level of phylum Verrucomicrobia while FMT had no such effect (Figure S2B). The species *Akkermansia muciniphila* mainly contributed to the change in levels of the Verrucomicrobia phylum (Figures S2B,E).

Within Firmicutes at genus level, FMT dramatically altered the relative abundance of the genus *Ruminococcus*. Y74 treatment changed the levels of genera *Clostridium* and *Oscillospira*. HT121 mainly changed the relative abundance of *Coprococcus* and *Roseburia*. Within Bacteroidetes, FMT dramatically altered relative abundance of the *Parabacteroides* genus. Y 74 treatment changed the abundance of the *Prevotella* genus, while HT121 treatment mainly changed the relative abundance of the *Bacteroides* genus (Figure 3A).

### FMT, *Lactobacillus* Y74, and HT121 Treatment Affected the Fecal Microbiota Composition at the Levels of the Family and Genus

**Figure 4:**
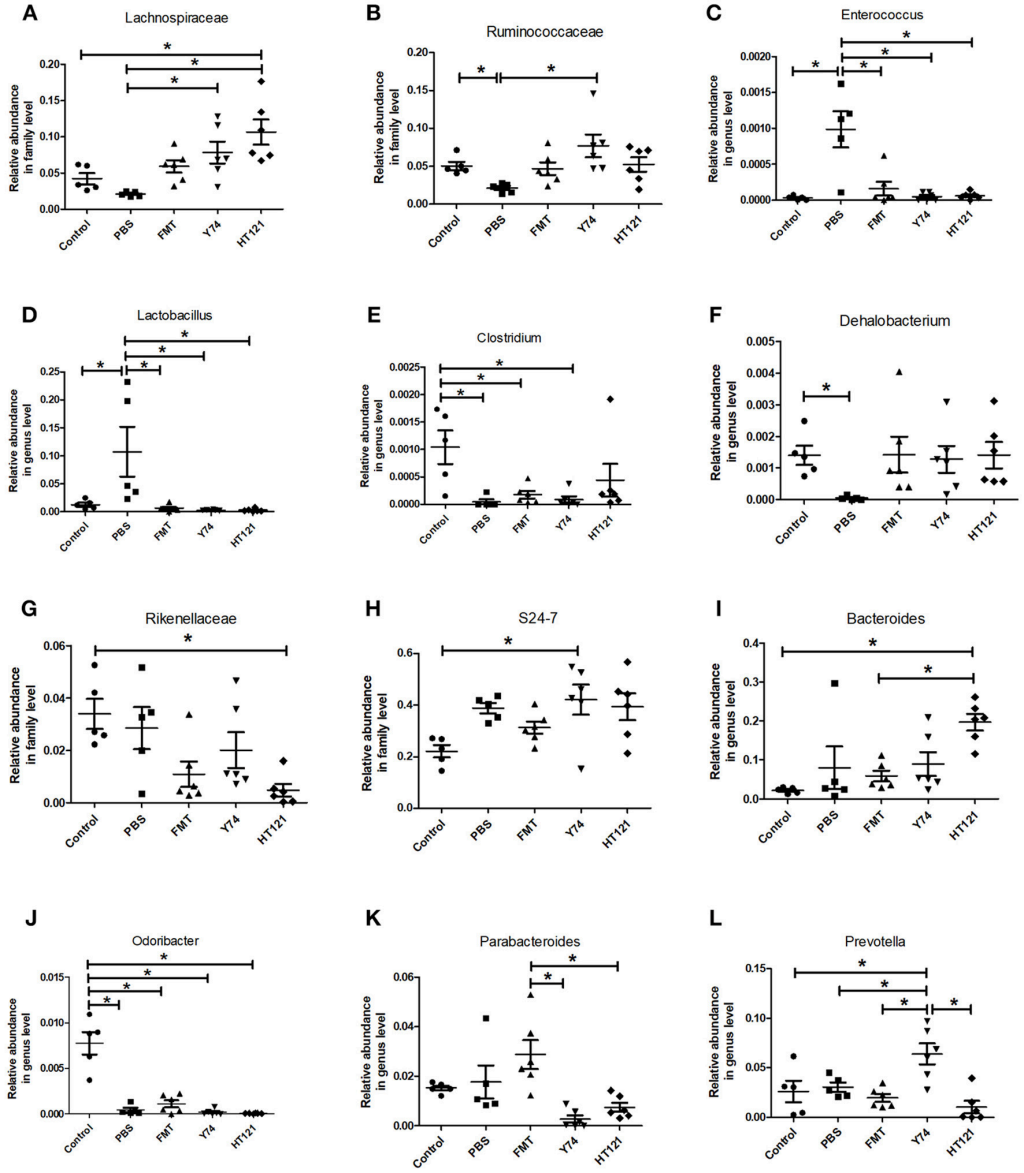
Comparison of the relative abundance of the most differential fecal microbiota in Control, PBS, FMT, Y74, and HT121 group. **(A**,**B)** represented relative abundances at family level of Lachnospiraceae and Ruminococcaceae; **(C–F)** represented relative abundances at genus level of *Enterococcus, Lactobacillus, Clostridium*, and *Dehalobacterium*, respectively. **(G,H)** Represented relative abundances at family level of Rikenellaceae and S24-7; **(I–L)** represented relative abundance in genus level of *Bacteroides, Odoribacter, Parabacteroides*, and *Prevotella*, respectively. *P < 0.05.

The level of the Lachnospiraceae family in PBS group was significantly lower than those in Y74, and HT121 groups. The abundance of the family Lachnospiraceae was even higher in the HT121 group than in the control group, suggesting that *Lactobacillus* Y74, and HT121 can promote multiplication of the Lachnospiraceae family (Figure 4A). The abundance of the Ruminococcus family was lower after VRE infection following antibiotic pretreatment, and only Y74 treatment reversed the relative abundance of the Ruminococcus family to similar levels seen in control mice (Figure 4B). Within the Bacillus family, antibiotic pretreated VRE infection increased the levels of the genera *Enterococcus* and *Lactobacillus* compared with uninfected mice, while FMT, Y74, or HT121 significantly reduced the levels of *Enterococcus* and *Lactobacillus* to the levels similar to those seen in uninfected mice (Figures 4C,D). Antibiotic pretreated VRE infection decreased the relative abundance of *Clostridium* and *Dehalobacterium* genera, and HT121 treatment reversed *Clostridium* genus to the levels seen in control mice, while no such effect was observed with FMT or Y74 treatment (Figure 4E). All three treatments (FMT, Y74, HT121) could restore the *Dehalobacterium* genus to levels similar to those in the control mice (Figure 4F).

The relative abundance of the Rikenellaceae family in the HT121 group was significantly lower than that of the control group, and there were no significant differences among PBS, Y74, FMT, and control groups (Figure 4G). The family S24-7 was significantly higher in the Y74 group than in the uninfected group (Figure 4H). The levels of genus *Odoribacter* in all infected groups were significantly lower than those of the control group (Figure 4J). There was no significant difference in relative abundance of *Bacteroides* among control, PBS, and the FMT group, while the relative abundances in the HT121 group was significantly higher than those in control and FMT groups (Figure 4I). VRE infection had no effect on the levels of the genus *Parabacteroides*; FMT treatment increased, while Y74 or HT121 treatment decreased the levels of *Parabacteroides* (Figure 4K). Neither VRE infection nor treatments changed the levels of *Prebotella*, except Y74 treatment which increased its abundance (Figure 4L). To validate the reliability of 16S rRNA sequencing, *Enterococcus* and *Lactobacillus* were selected for assessment by real time quantitative PCR. The copies of *Enterococcus* and *Lactobacillus* were consistent with their relative abundance (Figures S4A,B).

### Identification and Analysis of Differentially Expressed Genes (DEGs) Induced by FMT, *Lactobacillus* Y74, and *Lactobacillus* HT121

**Figure 5:**
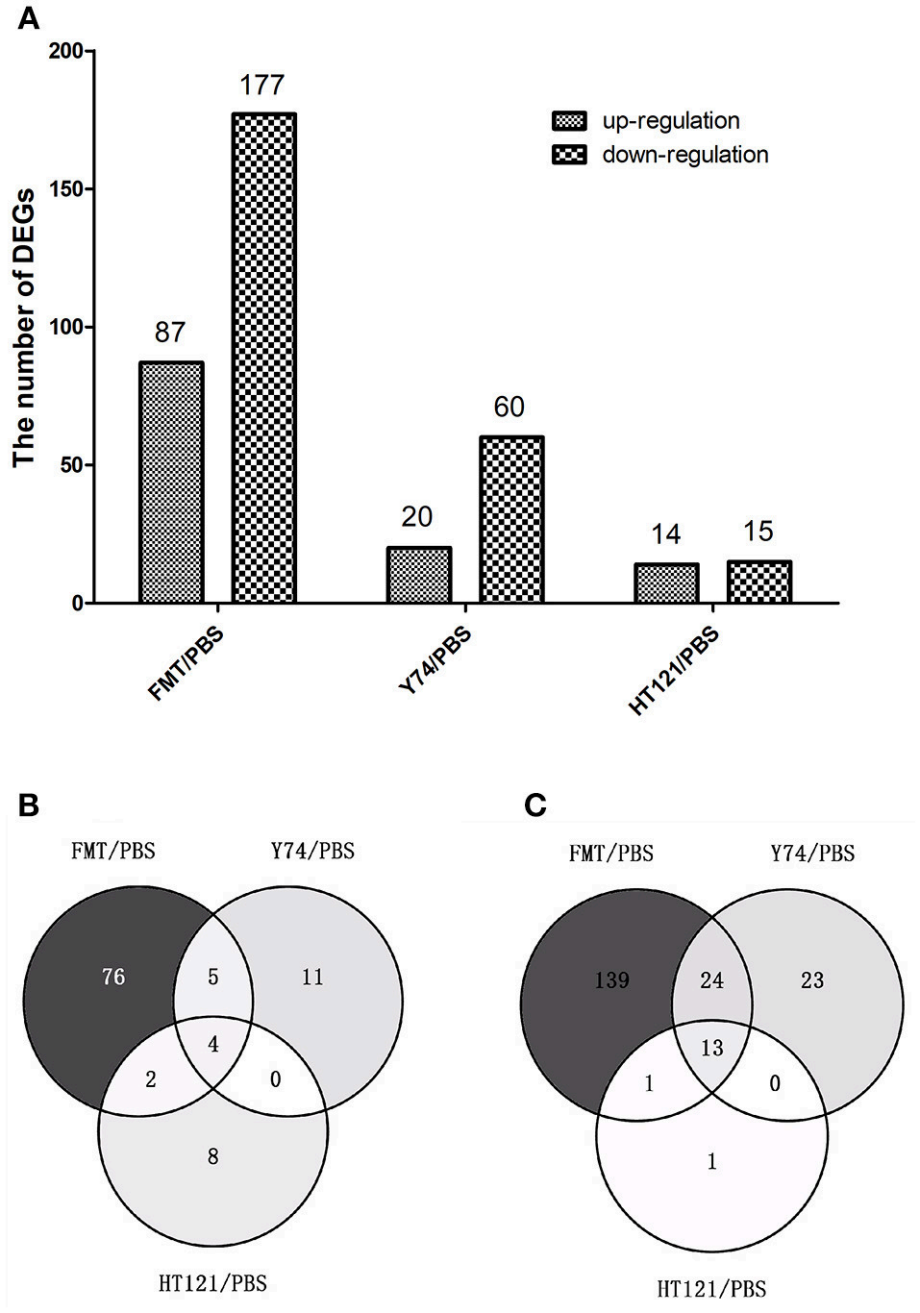
The number and comparison of up-regulated and down-regulated Differentially Expressed Genes in FMT/PBS, Y74/PBS and HT121/PBS. **(A)** represents the numbers of up-regulated and down-regulate DEGs, **(B)** represents the comparison of up-regulated DEGs in Venn diagram; **(C)**represents the comparison of down-regulated DEGs in Venn diagram.

To further explore the mechanisms for the VRE-lowing effect of different treatments, we conducted RNA-seq analysis of host gene expression. Compared with the PBS control treatment, FMT, Y74, and HT121 treatment induced upregulation of 87, 20, and 14 genes, and downregulation of 177, 60, and 15 genes, respectively (Figure 5A; Tables S2–S4). Among the up-regulated genes induced by treatments of FMT, Y74, and HT121, there were only four common genes (Fam177a, Slc15a2, Gm933 and 1810046K07Ri) (Figure 5B). There were 13 common genes among the down-regulated genes induced by all three treatments and these genes were mostly related to defense (Figure 5C). GO functional annotation of the DEGs indicated that the up- and down-regulated genes could be classified into three major categories: biological processes, molecular function, and cellular component. Most of the DEGs belonged to the function categories of biological process including biological regulation, cellular process, multicellular organismal process, metabolic process, regulations of biological process, response stimulus, and single-organism process. Some DEGs were related to binding and the catalytic activity in the molecular function while some DEGs were involved in cell part, macromolecular, membrane, and organelle in the cellular component. In general, FMT had more DEGs in the functional categories of biological process compared with the treatment group of Y74 or HT121 (Figures S3A–C).

**Figure 6:**
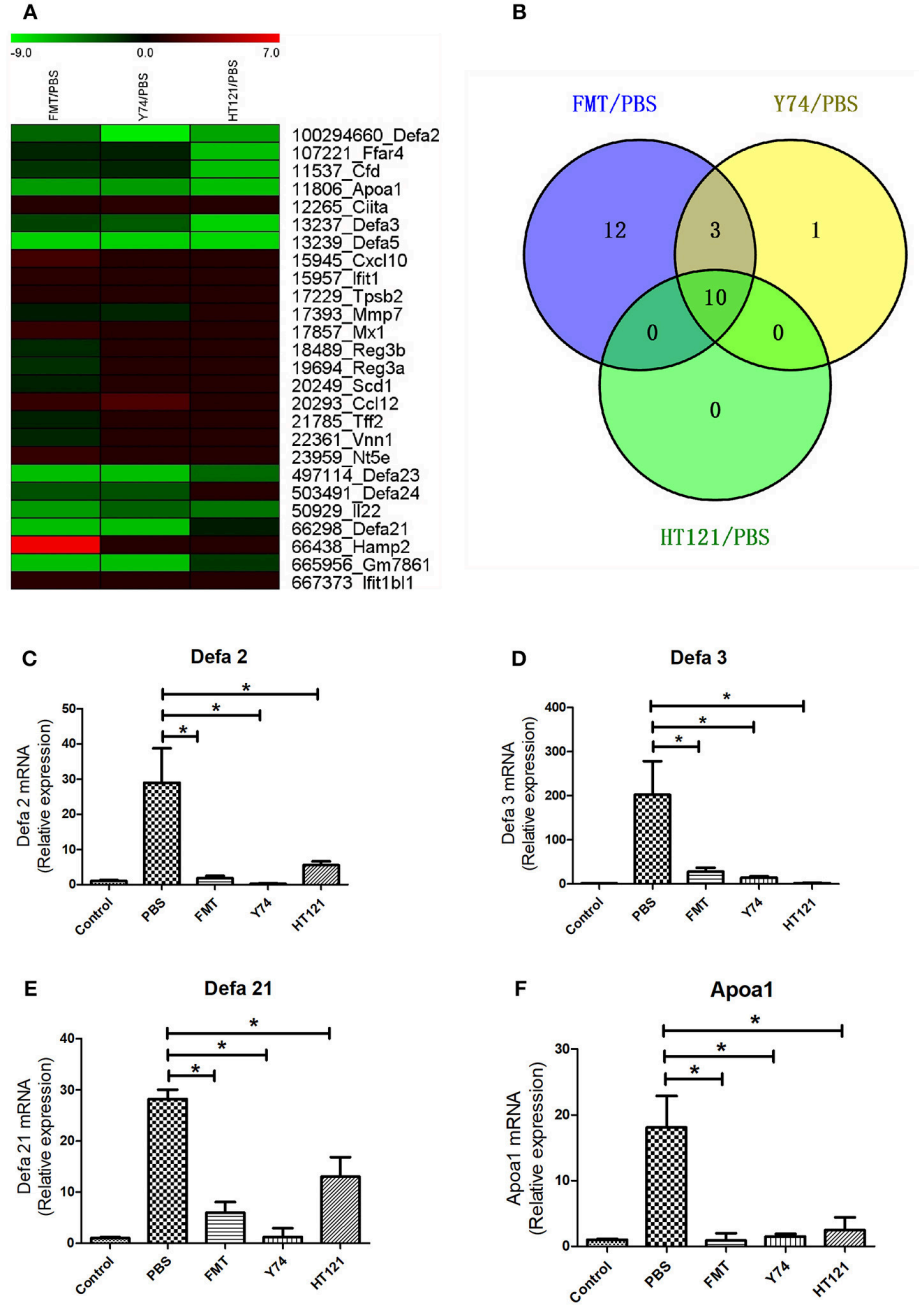
The heat map of up-regulated and down-regulated expression of defense-related genes in FMT/PBS, Y74/PBS, and HT121/PBS and the relative expression of *defa2, defa3, defa21* and *Apoa1*. **(A)** represents the heat map of up-regulated and down-regulated gene expression; **(B)** represents comparison of up-regulated and down-regulated gene expression in Venn diagram; **(C–F)** represent relative expressions of *defa2, defa3, defa21*, and *Apoa1* mRNA, respectively. Data are means ± SD, *n* = 3 mice. *P < 0.05.

Many studies showed that defense response played a critical role in eliminating VRE. Among 738 up-regulated genes and 29 down-regulated genes, we identified a total of 26 genes involved in the defense function. Heat map analysis showed 7 up-regulated DEGs and 18 down-regulated DEGs in FMT group, 2 up-regulated DEGs and 12 down-regulated DEGs in Y74 group, and 10 down-regulated genes in HT121 group (Figures 6A,B). In particular, decreased expression of Apoa1 and defensins was validated by quantitative real-time PCR (Figures 6C–F). There were 10 common genes in the three groups and all of them were down-regulated genes (Figure 6B). Fecal transplantation had a more dramatic influence on mice in the down-regulated defense genes than Y74 and HT121 treatments.

## Discussion

Reducing the colonization and shedding of VRE is important for preventing transmission between patients. Our study demonstrated that treatments with FMT, *Lactobacillus murinus* Y74 or *Lactobacillus plantarum* HT121 all significantly reduced the density of intestinal VRE colonization in mice after 3 days of treatment, and the *Lactobacillus plantarum* HT121 can affect the VRE counts in the earlier days. Our results are consistent with a previous animal study reporting that the *Lactococcus lactis* MM19 strain could prevent VRE overgrowth (Millette et al., 2008). Together, our results suggest that two *Lactobacilli* may have the probiotic potential to reduce VRE colonization in the gut of VRE-infected mice with an efficacy similar to FMT.

Our results indicate that *Lactobacilli* treatments more quickly restored the abundance of the Firmicutes than FMT did. The families of Lachnospiraceae and Ruminococcaceae, which belong to the Firmicutes Phylum, were restored to normal levels after mice were treated with *Lactobacillus* or FMT. Several members of these two families have been shown to produce short chain fatty acids (SCFAs) (Antharam et al., 2013), modulate formation of secondary bile acids (Devlin and Fischbach, 2015), and suppress *Clostridium difficile* colonization (Seekatz et al., 2018). Therefore, we speculate that the increased levels of the Lachnospiraceae and Ruminococcaceae families may contribute to decreased VRE colonization. In the current study, only HT121-treated mice had the abundance of the *Clostridium* genus restored to the normal levels. Studies have shown that some strains of *Clostridium* can induce the expression of FoxP3+ Treg cells in the intestine to inhibit inflammation (Gagliani et al., 2014), and some strains of *Clostridium* can produce butyrate (Van den Abbeele et al., 2013). Thus, in our study, the observed difference in *Clostridium* levels between the two *Lactobacilli* groups suggest that the two strains may decrease the number of VREs in the gut through a different mechanism. In the current study we also observed that the proportion of the *Enterococcus* genus in VRE-infected mice treated with HT121, Y74, or FMT was significantly lower than that in the VRE-infected mice treated with PBS, which may be due to the decreased number of VREs which belongs to the Enterococcus family. Interestingly, we observed that mice orally administered with 5 × 10^8^ CFU of *Lactobacilli* for 3 days had levels of *Lactobacillus* in feces similar to those seen in uninfected mice, which implies that the exogenous non-homogeneous *Lactobacillus* may not settle in the intestine and thus its VRE-lowing function may not be due to the colonization resistance of *Lactobacillus*.

There was no difference in the levels of Bacteroidetes between FMT treatment group and the control group, but PBS- and *Lactobacilli*-treated mice had significantly higher levels of Bacteroidetes than uninfected mice. Our results showed that FMT treatment could restore the levels of Bacteroidetes more quickly than other treatments. The genus of *Bacteroides* can produce propionate (Van den Abbeele et al., 2013; Walker and Parkhill, 2013) and are involved in the transformation of bile acids (Nicholson et al., 2012). The abundance of *Bacteroides* was high in IBD patients (Munoz et al., 2016). In the current study, abundance of *Bacteroides* was not restored to normal levels by HT121, while a increasing trend was observed in the other treatment groups. A possible explanation for this is that the VRE in the intestine may cause inflammation, and inflammation is positively correlated with the abundance of *Bacteroides*. A previous study showed that colitis was associated with an increased number of the *Prevotellaceae* genus (Elinav et al., 2011). Since only the Y74 treatment group induced high abundance of *Prevotellaceae*, it is possible that the two *Lactobacilli* strains differentially affected gut microbiota.

The Enterobacteriaceae family was significantly higher in PBS-treated, infected group than all the other groups, suggesting that FMT and two *Lactobacilli* could all inhibit Enterobacteriaceae. Antibiotic-associated infection can increase the numbers of the Enterobacteriaceae family (Lichtman et al., 2016), which has thus become one of the indicator phylotypes of colitis (Berry et al., 2012). The inflammatory host response can selectively enhance growth of the Enterobacteriaceae family (Winter et al., 2013). Certain Enterobacteriaceae can produce Extended-spectrum β-lactamase (ESBL), which can resist carbapenem, subsequently called *Carbapenem-Resistant Enterobacteriaceae* (CRE) (Haidar et al., 2017). Since we found that the two strains *Lactobacilli* and FMT decreased the abundance of the Enterobacteriaceae family to normal levels, they may play an important role in maintaining intestinal homeostasis.

The levels of phylum Verrucomicrobia in the Y74 and HT121 groups were significantly lower than those in the control and FMT group. Results of 16S rRNA analysis indicated that *Akkermansia municium* contributed to these differences. *Akkermansia municium* degrades intestinal mucosa and produces a class of oligosaccharides and SCFAs which participate in various metabolisms (Everard et al., 2013; Lukovac et al., 2014). It has been reported that *Akkermansia municium* negatively correlates with inflammation (Anhe et al., 2015). An increased proportion of *Akkermansia municium* helps to inhibit inflammation. Both FMT and *Lactobacilli* can significantly reduce the number of VRE, however, their effects on intestinal microflora may be different.

Previous studies have shown that increasing the level of RegIIIγ is effective for decreasing VRE infection (Brandl et al., 2008). Flagellum was also shown to stimulate intestinal epithelial cells and Paneth cells to express RegIIIγ through TLR5, and promote the clearance of VRE (Kinnebrew et al., 2010). Based on these studies, we speculate that the bactericidal substances such as bacteriocin, defensin, and Reg III may be higher in mice treated with probiotics, compared to those in the PBS group. However, the data of RNA-seq and real-time PCR in fact showed the opposite results, i.e., defensin, IL22, and RegIIIγ in treatment groups were significantly lower than the PBS control group. VRE can stimulate the production of defensin, IL22, and regIII by the mouse intestine, which could possibly be explained by the intervention by FMT or *Lactobacilli* rapidly decreasing the number of intestinal VREs leading to lower levels of defensin, IL22, and regIII expression. We found significantly lower mRNA expression of APOa1 in intervention treatment groups compared to the PBS group. Our results are consistent with the defense function of APOa1 (Tomazic et al., 2014). In the colon RNA-seq analysis, we found that FMT caused altered expression of more genes compared to *Lactobacilli*, probably because of the higher diversity of bacteria and the more complex metabolic processes in the feces induced altered expression of more genes. As for the smaller number of genes expressed in the HT121 group compared to the Y74 group, we speculate that the two trains may act with different mechanisms, e.g., HT121 may act earlier to decrease the number of VREs, which induce altered expression of more genes.

In summary, optimal intervention of VRE colonization in the gut should decrease the number of VREs in the gut microbiota and restore the gut flora to its normal composition in the shortest time possible, thus minimizing the activation of host defense genes. We found that administration of *Lactobacillus murinus* Y74 and *Lactobacillus plantarum* HT121 quickly and effectively decreased VRE colonization in the intestine of VRE-infected mice, and restored diversity of intestinal microbiota. *Lactobacilli* could promote restoration of the Firmicutes Phylum to normal levels more rapidly than FMT. More genes related to immune defense were down-regulated by administration of FMT, or *Lactobacillus* Y74 and HT121, which may help restore intestinal homeostasis. However, our conclusions are based on ampicillin-facilitated VRE infection in the mouse model, which has a short period of VRE colonization and does not accurately mimic the VRE colonization in humans. Additionally, the ampicillin treatment likely had an impact on the recovery of microbiota, which may confound the effect of FMT or *Lactobacilli*. Therefore, the translational value, i.e., whether FMT or *Lactobacillus* Y74 and HT121 can be effective in treating VRE-infected patients requires validation in future studies.

## Author Contributions

XL and ZR conceived and designed the experiments and wrote the paper. XL, LS, SZ, YtH, YX, and QC performed the experiments. XL and YmH analyzed the data. XL, LS, and ZR discussed the results.

### Conflict of Interest Statement

The authors declare that the research was conducted in the absence of any commercial or financial relationships that could be construed as a potential conflict of interest.

## Supplementary Material

Figure S1 Comparison of the composition ratio of fecal microbiota in the level of Phylum, Family, Genus and Species. **(A–D)** showed relative abundances of the fecal microbiota in the level of Phylum, Family, Genus and species, respectively. “o_” represented order, “f_” represented family, “g_” represented genus, “s_” represented species, respectively.

Figure S2 Comparison of the relative abundance of fecal microbiota of control, PBS, FMT, Y74, and HT121 group in Proteobacteria and Verrucomicrobia Phylum. **(A,B)** represent the Proteobacteria and Verrucomicrobia Phylum relative abundance in five groups; **(C)** was relative abundance in family level of Enterobacteriaceae; **(D,E)** was relative abundance in genus level of *Sutterella* and *Akkermansia*, respectively. ^*^*P* < 0.05.

Figure S3 The number of up-regulated and down-regulated difference expression genes of FMT/PBS, Y74/PBS and HT121/PBS in biological process **(A)**, molecular function **(B)** and cellular component **(C)**.

Figure S4 The copies of *Enterococcus* and *Lactobacillus* genus per 1 g feces as determined by real time quantitative PCR. **(A,B)** represent *Enterococcus* and *Lactobacillus* genus, respectively. Data are means ± SD, *n* = 6 samples. ^*^*P* < 0.05.
