# Supplementary figures and images for: Two Strains of Lactobacilli Effectively Decrease the Colonization of VRE in a Mouse Model
Source: Front Cell Infect Microbiol. 2019 Jan 30;9:6. doi: 10.3389/fcimb.2019.00006 (PMC6363661; doi:10.3389/fcimb.2019.00006)

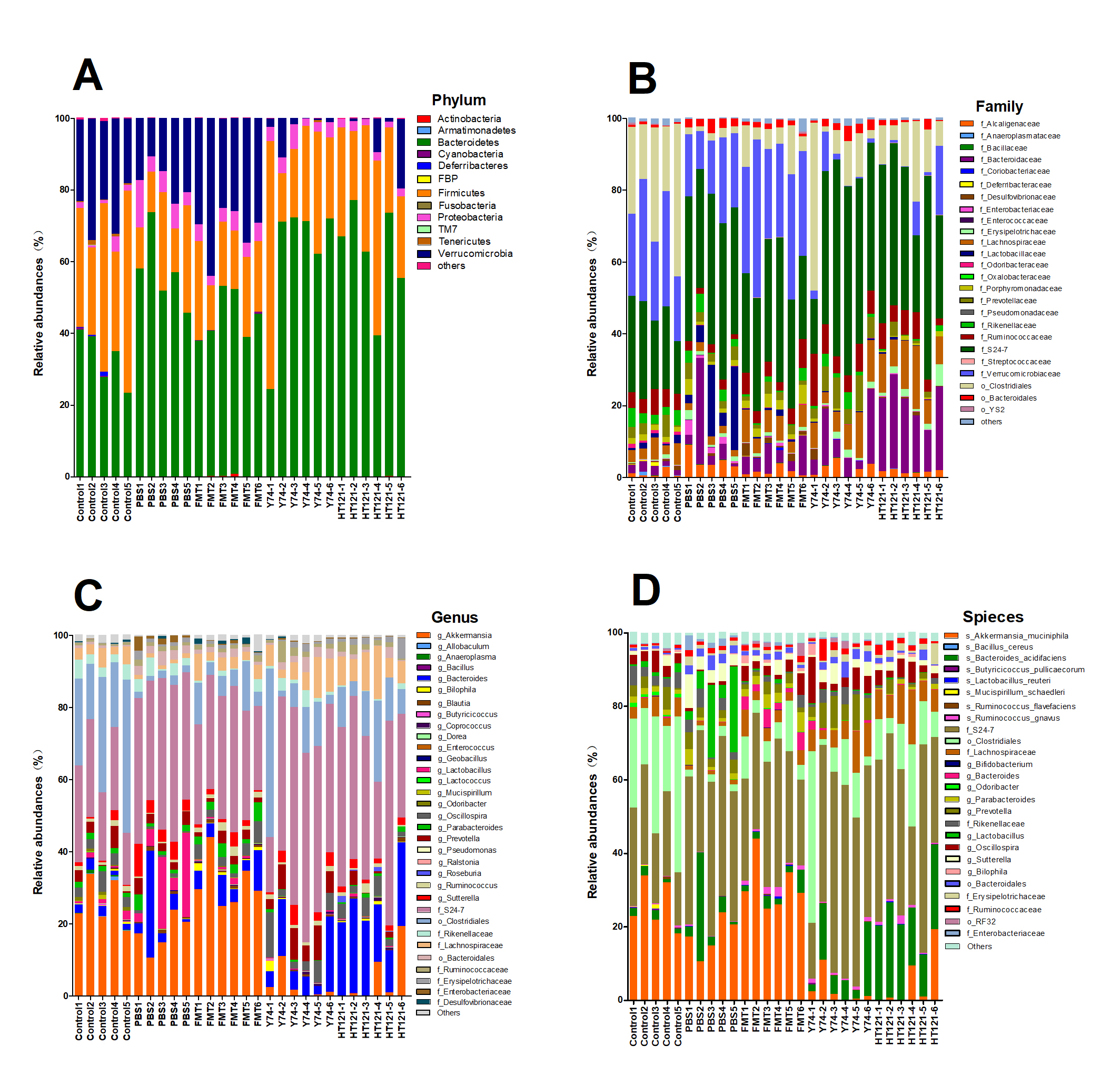

Supplement: Figure S1 — Comparison of the composition ratio of fecal microbiota in the level of Phylum, Family, Genus and Species. (A–D) showed relative abundances of the fecal microbiota in the level of Phylum, Family, Genus and species, respectively. “o_” represented order, “f_” represented family, “g_” represented genus, “s_” represented species, respectively. [file Image_1.JPEG]

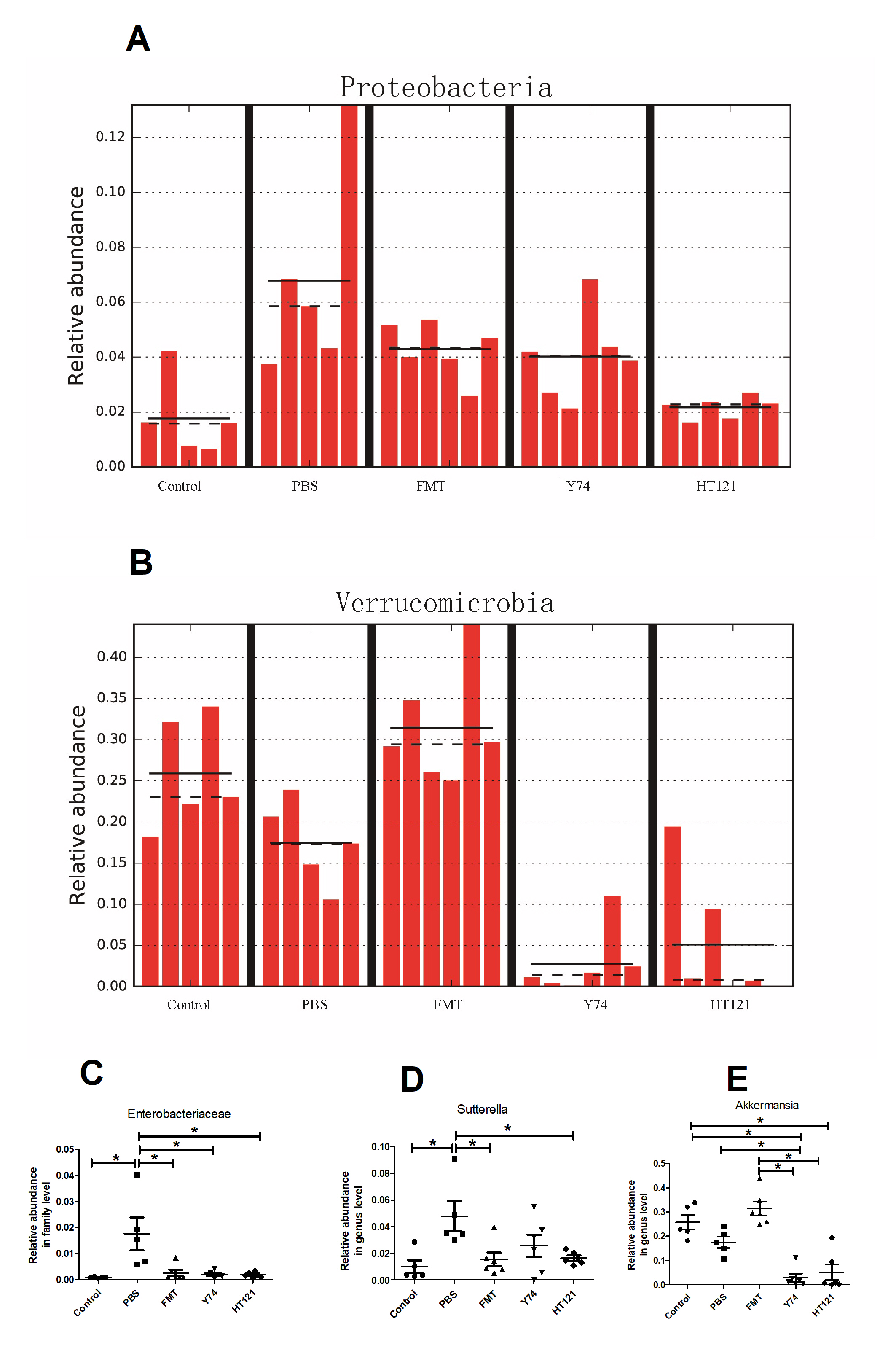

Supplement: Figure S2 — Comparison of the relative abundance of fecal microbiota of control, PBS, FMT, Y74, and HT121 group in Proteobacteria and Verrucomicrobia Phylum. (A,B) represent the Proteobacteria and Verrucomicrobia Phylum relative abundance in five groups; (C) was relative abundance in family level of Enterobacteriaceae; (D,E) was relative abundance in genus level of Sutterella and Akkermansia, respectively. *P < 0.05. [file Image_2.JPEG]

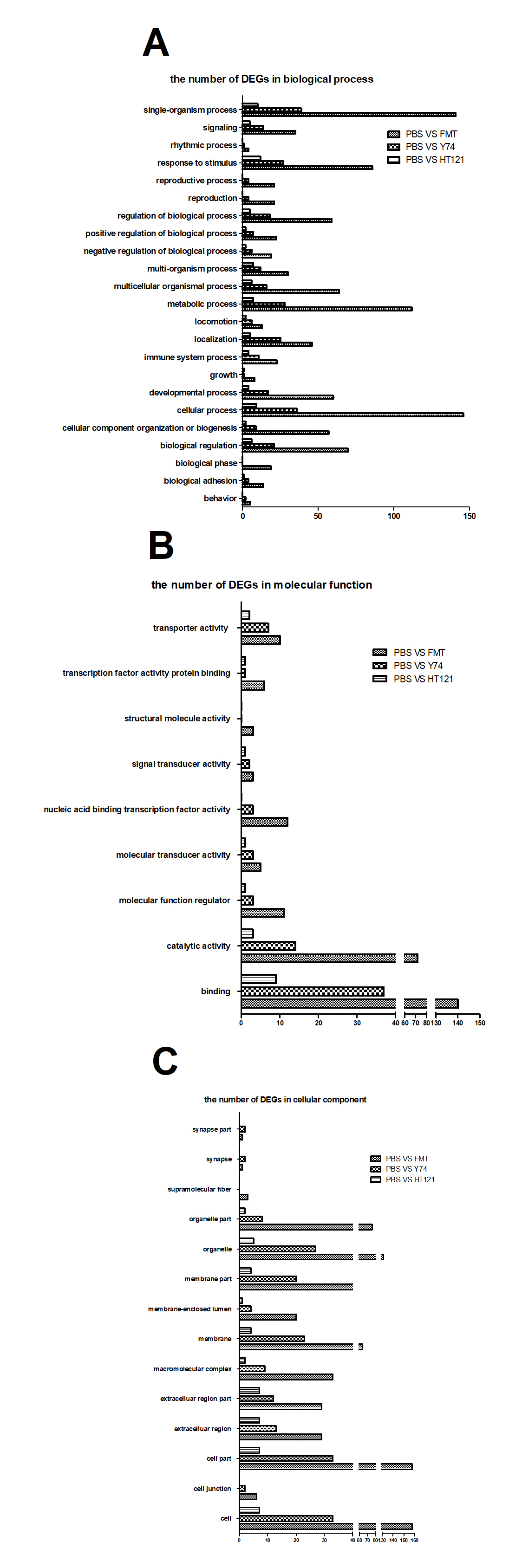

Supplement: Figure S3 — The number of up-regulated and down-regulated difference expression genes of FMT/PBS, Y74/PBS and HT121/PBS in biological process (A), molecular function (B) and cellular component (C). [file Image_3.JPEG]

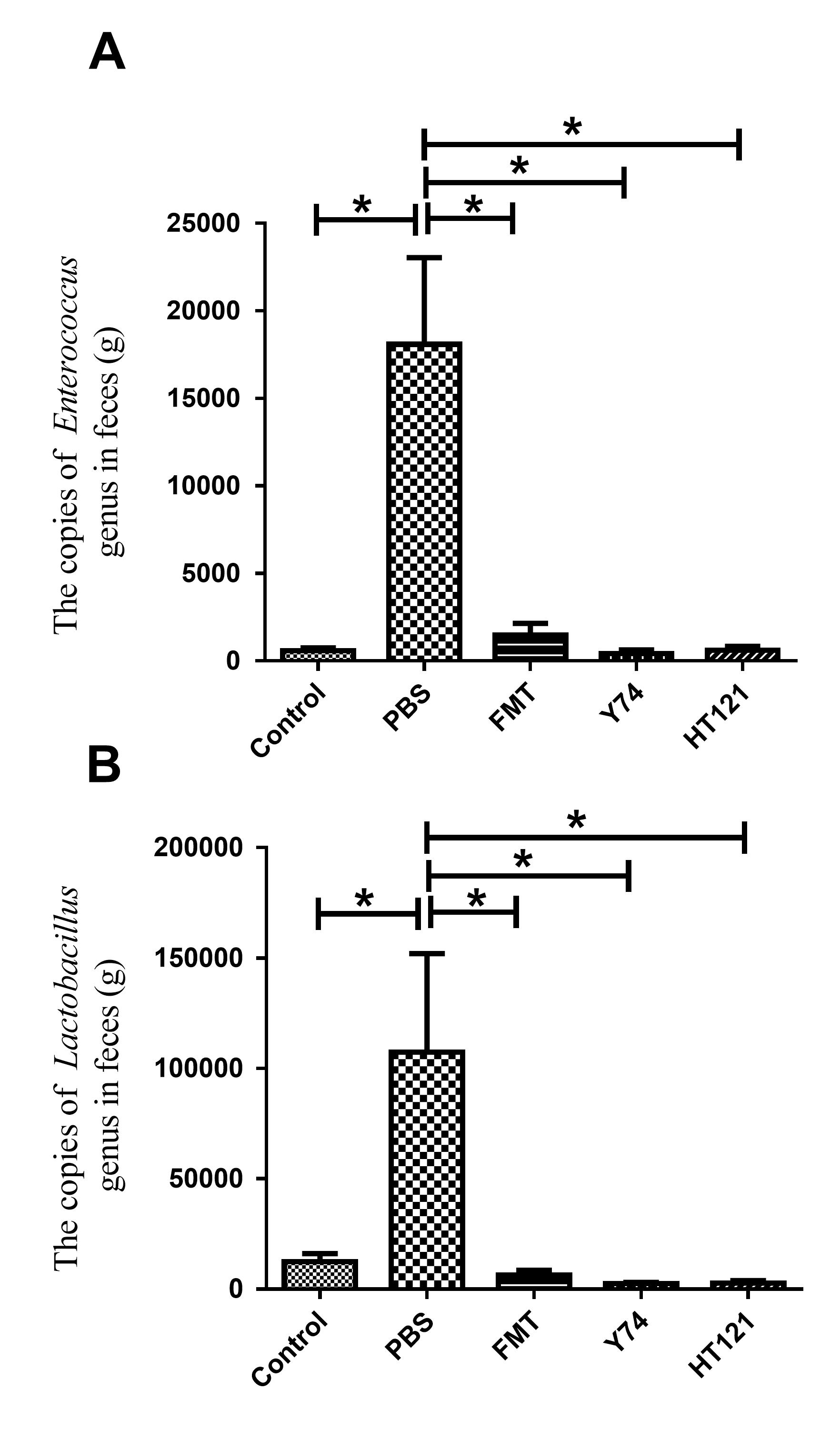

Supplement: Figure S4 — The copies of Enterococcus and Lactobacillus genus per 1 g feces as determined by real time quantitative PCR. (A,B) represent Enterococcus and Lactobacillus genus, respectively. Data are means ± SD, n = 6 samples. *P < 0.05. [file Image_4.JPEG]
